# Repeating the errors of the past: the hazards of a commercial human milk industry

**DOI:** 10.1038/s41390-023-03004-3

**Published:** 2024-01-09

**Authors:** Neena Modi

**Affiliations:** Professor of Neonatal Medicine, Imperial College London, Section of Neonatal Medicine, School of Public Health, Chelsea and Westminster Hospital campus, 369 Fulham Road, London, UK

## Introduction

Recent years have seen the emergence of several for-profit human milk companies around the world. These pay lactating women for milk, which they process and sell as a variety of products. Typical payments are around £3/100 ml; typical prices are around £15 per 100 ml for a basic human milk feed rising to £100–£220 for fortifier sufficient for 100 ml of human milk. The rise of a commercial human milk industry raises medical practice, health services, patient safety, and ethical concerns, and may prove as hazardous to infant wellbeing as the formula milk industry.

## Is human donor milk needed?

Wet-nursing has been practised by all societies throughout time. When no safe alternatives existed, this was life-saving if a mother died. It was also an affectation of rich women who chose not to feed their babies and could afford a wet nurse. Historical records provide vivid descriptions of the tragic consequences to infants fed alternatives that included animal milks, and liquid squeezed from bread or grain soaked in water (respectively known as pap and panada). The rise of the formula industry expanded available options, but their first products also posed serious health risks because they were poorly digestible, of inappropriate composition, and carried a high risk of infection from unhygienic preparation or storage.

Today, there is still need for supplementary feeds. Human milk remains an option, but pasteurisation is recommended to minimise the risks of transmission of infectious agents. Safe formulae are also now available, including products designed for very preterm babies for whom the need for supplementary feed options is a particular issue. These infants typically spend many weeks in a neonatal unit. Their developmental immaturity means they are unable to suckle, hence their mothers are faced with having express milk for a prolonged period which is difficult and stressful. The most recent UK National Neonatal Audit found only 60% of babies born very preterm were receiving any own mother’s milk at discharge.^1^

## What are the benefits of pasteurised human milk?

Many organisations recommend pasteurised human donor milk as the option of choice if milk from a baby’s own mother is unavailable. The World Health Organisation (WHO) published guidelines in 2011 recommending that low birthweight infants in low-and middle-income countries who cannot be fed their own mother’s milk should receive human donor milk, but accompanied this with the qualification that facilities must be available for safe and affordable human milk banking, in recognition that the cost of providing safe supplies of donor milk is not trivial.^2^ The American Academy of Pediatrics and European Society for Paediatric Gastroenterology, Hepatology and Nutrition also support use of pasteurised human donor milk as the supplementary feed of choice. However, clinical trials to-date do not show benefit when pasteurised human donor milk is compared with formula as a supplement to own mother’s milk.

It is plausible that fresh own mother’s milk reduces the risks of infection and the feared, acquired gastrointestinal inflammatory condition, necrotising enterocolitis (NEC), as human milk evolved primarily for anti-infective purposes. Many clinicians believe pasteurised human milk is similarly beneficial, but corroboratory evidence is lacking. The current Cochrane review identifies 12 randomised controlled trials involving 1879 infants that compared pasteurised human donor milk and formula.^3^ Data on NEC from 9 trials in which the comparison was as sole diet or supplement to own mother’s milk show a higher risk in the formula group (Relative Risk (RR) 1.87; 95% CI 1.23 to 2.85) which is often cited as justification for widespread adoption of pasteurised human donor milk and expansion of human milk banking. However, when sole and supplemental diet trials are considered separately the authors find a reduction in NEC only in sole and not in supplementary diet comparisons. They also find no statistically significant differences for either sole or supplementary comparisons in outcomes that would provide important corroboration of benefit (mortality, invasive infection, neurodevelopment). The authors sound other notes of caution. Seven of the 12 trials took place in the 1970s and 80 s when the patient population was very different from that of today. The sample sizes lack power to detect important effects, methodological quality was poor, and medically managed necrotising enterocolitis was included in the outcome, though this is an imprecise diagnosis highly liable to ascertainment bias. The Cochrane reviewers conclude further research is needed to establish the effects of feeding with formula or donor milk when the expressed milk of a preterm infant’s mother is not available.

Failing to distinguish own mother’s milk from pasteurised human donor milk risks implying equivalence which is misleading. Pasteurised human donor milk is not equivalent to own mother’s milk as pasteurisation reduces or destroys many non-nutritive components such as immunologically active agents, growth factors, and probiotic species. A mother’s motivation to express may be reduced if she mistakenly believes pasteurised human donor milk is equivalent to her milk. Additionally, a systematic review and meta-analysis showed no difference in exclusive breastfeeding at hospital discharge in very preterm infants after the introduction of pasteurised human donor milk and a decrease in some settings.^4^

## Safety considerations

Though the consideration is troubling to many, there are legitimate concerns regarding the possibility of harm from pasteurised human donor milk. Pooled human milk has variable and low nutrient density which may be inadequate to support the growth and brain development of very preterm babies, and pasteurisation reduces or destroys many anti-infective components. Randomised controlled trials show growth is slower with pasteurised human donor milk, even when nutrient enriched.^3^ A Canadian trial comparing nutrient enriched pasteurised human donor milk or preterm formula as a supplement to own mother’s milk, showed more children randomised to donor milk with neuro-impairment at 18 months (27.2% v 16.2%; adjusted risk difference 10.6% [95% CI 1.5%, 19.6%]). The donor milk group also had a worse mortality/morbidity index (43% v 40%).^5^ We conducted an analysis of UK population data employing techniques designed to minimise confounding and compared survival to 34 weeks postmenstrual age without surgery for NEC in very preterm babies who had received feeds of own mother’s milk supplemented with either pasteurised human donor milk or formula. We found almost 10% lower survival without NEC surgery in the group receiving the pasteurised human donor milk supplement (Adjusted Risk Difference −9.8% [−11.4, −8.2]).^6^ This was most pronounced in babies born at 24 weeks gestation (−51.5% [−58.8, −44.3]) and decreased with increasing gestational age, with minimal difference for babies born at 31 weeks. NEC surgery, NEC-related deaths and all-cause mortality were also all higher in the group receiving pasteurised human donor milk supplements.

## Health services considerations

Pasteurised human donor milk costs around £90–150/litre compared to a cost of around £5/litre for formula. This is an important issue for health services. Establishing and running a human milk bank are major undertakings, hence many are managed as charities or not-for-profit social enterprises. The regulation of human milk banks is largely voluntary though there are repeated calls for stronger guidance and for considering human milk a biological fluid that should be regulated in the same way as blood and tissue.^7^

Human milk companies claim their products are cost-effective primarily based on reductions in NEC, and their prices are therefore justified. However, the Cochrane review comparing human and cow milk‐derived fortifier in preterm infants identified only one randomised controlled trial.^8^ This enrolled 127 infants and showed no differences in NEC or other outcomes. A yet unpublished trial (NCT03797157) conducted in Sweden, compared 228 infants randomised to human or cow milk fortifier and also found no differences in NEC and other outcomes. Until such time as there is evidence of the efficacy of these products in improving important functional health outcomes, claims of cost-benefit must be considered highly questionable.

## Clinician bias

Clinicians of all disciplines are human beings with personal beliefs and biases. Prior to the emergence of the concept of evidence-based medicine, it was appropriate for a physician to rest content if their conscience told them they were doing their best for their patient according to their experience and knowledge; indeed, to do so was enshrined in the Hippocratic Oath. Today however, a physician must look to the evidence and the quality of that evidence, and set aside their own beliefs, when recommending treatments.

In our focus group sessions with clinicians, several candidly articulated the anxiety they felt when confronted with a conflict between the evidence and their personal beliefs.^9^ For many, this cognitive dissonance results in rejecting participation in randomised controlled trials to address uncertainties and not offering their patients opportunity to participate. This choice not only imposes personal biases upon patients, but reflects a paternalistic attitude to care, in which the physician “knows best”. By so acting they have failed to uphold the four cardinal principles of medical ethics; the patient (in this case the parent) has been denied autonomy, the right to decide for themselves whether to participate in a clinical trial. The patient has been denied justice, opportunity to have a fair and equal chance of receiving the unknown optimum treatment through randomisation. The doctor has failed to act with beneficence, to give the patient a chance to receive the optimum treatment, and with non-malevolence, by imposing their personal beliefs. Realising that meaning well, may run counter to the cardinal principles of medical ethics can be painful but must be confronted if patients are to be protected against the dangers of non-evidenced care, and their right to autonomy, respected.

## Other ethical considerations

Commercial human milk companies market their outputs as nutritional products. Some are registered as dairy companies (e.g., NeoLacta), though their advertising implies health benefits, and their names reference medical science (e.g., Prolacta Bioscience (prolacta.com); NeoLacta Lifesciences (neolacta.com)). Neokare (neokare.co.uk) based in the UK, claims to have established the only pharmaceutical grade manufacturing facility for human milk in Europe. The aim of commercial suppliers of human milk is profit. They source milk from communities that are likely to be disadvantaged, and hence vulnerable to offers of payment. This has raised a variety of concerns, such that the mother’s own babies may be denied their milk. A US company was forced to back track following protests by Black women against their sourcing of milk from poor African American women,^10^ and the export to the US of human milk obtained from Cambodian mothers was halted by Government decree.^11^ Researchers have written about another for-profit human milk company that exports human milk from the US to Africa calling this an aid project, when it is an example of post-colonial “white-missionary” behaviour that has received wide condemnation.^12^ An Indian company’s plan to source milk from women without making any payment to them and sell this for a profit also received wide condemnation.^10^

It could be argued that individuals and organisations that promote pasteurised human donor milk as the optimum supplement for very preterm babies even through existing evidence is inconclusive, and there are risks of harm, have played into the hands of the commercial for-profit companies who are engaging in no less vocal campaigns directed at clinicians and parents, with the goal of persuading them that their expensive products benefit infant wellbeing. In the USA, families are also being persuaded to make claims against formula companies on the grounds that their products caused their babies to develop NEC.^13^

This adversarial situation based on claim and counter-claim rather than evidence does not serve families and patients well. Clinicians have a moral obligation to explain uncertainty with honesty. Pasteurised human donor milk *may* or *may not* be optimum as a supplement to own mother’s milk; formula *may* or *may not* be optimum; or they may be equivalent.

## Learning from past mistakes

Good science, and good medicine, are built upon objective evidence. Meaning well is not enough and often dangerous. The history of neonatal medicine has many examples of experts and organisations that recommended practices that were ultimately found to be harmful when put to the test of objective evaluation. These include thymic irradiation practised in the 1940s to reduce the risk of sudden infant death that led to many thousands of patients developing thyroid cancer, routine separation of mothers and babies well into the 1970s that caused huge psychological distress, prone sleeping that was ultimately found to increase, not decrease, cot death, immediate cord clamping, and high dose postnatal steroids. Clinical trials to examine the routine use of 100% oxygen for newborn resuscitation were vigorously opposed by many, but when finally conducted showed the practice to be harmful.

High-cost commercial human milk products are entering into use in an uncontrolled and non-evidenced manner, fuelled by aggressive and misleading marketing, tactics also used by the infant formula companies. Recognition of the harm this caused infants led to the 1981 WHO Code on the Marketing of Breastmilk Substitutes, and a recent report on digital marketing strategies for promoting breast-milk substitutes.^14^ Formula companies were so successful that mothers around the world were persuaded that their products were best for their babies. Are we witnessing history repeat itself? Are clinicians and mothers being persuaded that donor milk products are as good as, or almost as good as their own milk, or in the case of fortification, that their own milk is not good enough on its own? Will future clinicians and social scientists look back and say, “*how could they have made the same mistake again*”?

What is needed is for biases to be set aside in favour of resolving pressing uncertainties in rigorous randomised controlled trials. If pasteurised human donor milk is the optimal supplement, all very preterm babies should have equitable access, but if it is not, resources and energies should be directed elsewhere. The 2023 British Association of Perinatal Medicine “Framework for use of Donor Milk” highlights urgent need for research to identify the optimum supplement for own mothers’ milk.^15^ For other organisations, including WHO, and not-for-profit human milk banks to advocate similarly for research to resolve this longstanding, global uncertainty would be an honest stance that puts patient wellbeing first.
